# Coronary Artery Vasospasm Presenting as Non-ST Segment Elevation Myocardial Infarction in a Nigerian Female: A Case Report

**DOI:** 10.7759/cureus.101095

**Published:** 2026-01-08

**Authors:** Olurotimi J Badero, Bamikole Osibowale, Victor Ajayi, Olusegun D Alaga

**Affiliations:** 1 Interventional Cardiology, Iwosan Lagoon Hospitals, Victoria Island, Lagos, NGA; 2 Interventional Cardiology, Division of Cardio-Nephrology, Cardiac Renal and Vascular Associates, Jackson, USA; 3 Cardiology, Caribbean Tristate Heart Institute, Trinidad, TTO; 4 Department of Medicine, Lagos State University Teaching Hospital, Lagos, NGA

**Keywords:** coronary arteries, coronary artery angiography, coronary artery disease, nigeria, vasospasm

## Abstract

Coronary artery spasm (CAV) is characterized by transient vasoconstriction of normal or diseased epicardial coronary arteries, with a resultant reduction in myocardial perfusion, ischemia, infarction, or sudden cardiac death. Although the precise pathophysiologic mechanism of CAV remains unclear, vascular smooth cell contraction constitutes a core underlying feature.

A 46-year-old Nigerian woman with pre-existing hypertension was admitted following an episode of chest pain; subsequent elevation of cardiac biomarkers and electrocardiogram showing T-wave inversions in the anterolateral and inferior leads led to the diagnosis of non-ST-elevation myocardial infarction (NSTEMI). Coronary angiography was performed as part of an early invasive strategy. CAV was diagnosed by coronary angiography, which demonstrated diffuse constriction of the left anterior descending artery (LAD) in its mid and distal segments. Selective contrast injection into the left coronary system reproduced her typical chest pain symptom accompanied by transient ST-segment elevation in lead V1, which resolved spontaneously.

This case underscores that CAV is a pan-ethnic phenomenon that must be considered in the differential diagnosis of acute coronary syndrome across diverse patient populations. Provocative testing during angiography remains the diagnostic cornerstone. Management hinges on long-acting nitrates and calcium channel blockers for prevention, while avoiding non-selective beta-blockers. Prompt diagnosis and management, contingent upon clinical suspicion, are vital to preventing lethal complications. This report highlights the importance of recognizing CAV beyond its classic demographic associations to ensure equitable and effective care.

## Introduction

Coronary artery vasospasm (CAV), or Prinzmetal's angina, is a clinical syndrome characterized by transient, focal hyperconstriction of coronary arterial smooth muscle, leading to significant luminal narrowing and myocardial ischemia. This phenomenon, first described by Prinzmetal et al., is often accompanied by transient electrocardiographic changes, most notably ST-segment elevation or depression [1-3].

In cases of myocardial infarction with non-obstructive coronary arteries (MINOCA), CAV is a common underlying mechanism, with epidemiological studies demonstrating notable variations in prevalence across ethnicities and geographic regions [4]. Key demographic risk factors include male sex, age between 40 and 70 years, and Japanese or Taiwanese descent [5,6]. The risk of developing CAV is further elevated by modifiable factors, including smoking, hypertension, diabetes mellitus, dyslipidemia, and elevated high-sensitivity C-reactive protein (hs-CRP) levels [7]. Precipitating factors for acute episodes include alcohol consumption [8], cocaine use [9], prolonged emotional stress [10], extreme cold exposure, and various pharmacological agents including beta-blockers, anticholinesterases, and sympathomimetic or parasympathomimetic drugs [11].

The clinical presentation of CAV typically consists of rest angina, often with a circadian pattern manifesting in the early morning hours. Clinical severity varies widely, ranging from stable symptoms to acute coronary syndrome. The associated ischemic ECG changes reflect the anatomical severity of the spasm: ST-segment depression may accompany focal spasm in a minor artery or non-occlusive spasm in a major epicardial vessel, conversely transient ST-segment elevation typically indicates the complete or near-complete occlusion of a primary coronary artery [12]. Furthermore, a ventricular arrhythmia may also occur [13].

We report a case of CAV in a middle-aged Nigerian woman with hypertension, detailing the clinical presentation, management strategy, and outcome. This case highlights CAV in a relatively young woman with no smoking history. To our knowledge, this represents the first angiographically confirmed case of coronary vasospasm reported in the Nigerian literature.

## Case presentation

A 46-year-old female non-smoker with a history of hypertension presented with a three-day history of chest pain. The chest pain was typical, mid-sternal in location, exertional, and associated with nausea and shortness of breath.

**Figure 1:**
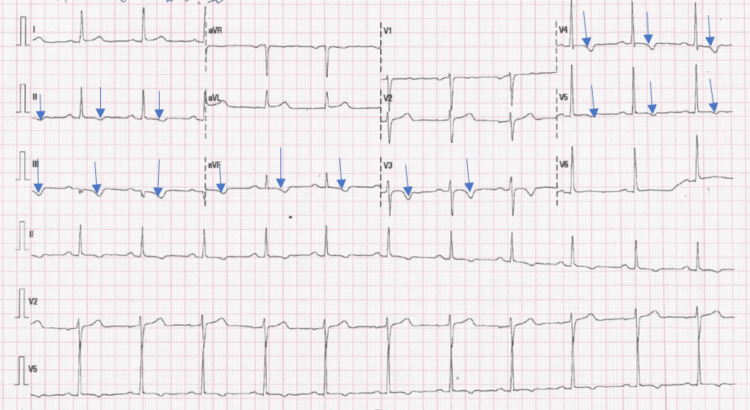
Electrocardiogram showing normal sinus rhythm with inverted T waves in the antero-lateral and inferior leads (blue arrows).

Vitals were normal and physical examination revealed no remarkable findings. Results of laboratory investigations are shown in Table 1. Her ECG revealed normal sinus rhythm with inverted T waves in the antero-lateral and inferior leads (Figure 1).

**Table 1:** Results of laboratory investigations. INR: international normalised ratio

TEST	VALUE	REFERENCE RANGE
Troponin I	0.63 ng/ml (0 hours); 2.50 ng/ml (6 hours); 2.60 ng/ml (12 hours)	0-0.04 ng/ml
Blood Count		
White cell count	5,260/µL	4,000-11,000/µL
Platelets	189,800/µL	150,000-400,000/µL
Haemoglobin	14.75 g/dL	12-16 g/dL
Renal Function Test		
Sodium	140 mmol/L	130-150 mmol/L
Potassium	3.5 mmol/L	3.5-5.5 mmol/L
Chloride	105 mmol/L	98-105 mmol/L
Bicarbonate	28 mmol/L	20-30 mmol/L
Urea	13 mg/dL	10-55 mg/dl
Creatinine	0.7 mg/dL	0.5-0.9mg/dl
Clotting Profile		
Prothrombin time	13.7 seconds	11.0-14.0 seconds
INR	1.17	0.8-1.20
Lipid Profile		
Total cholesterol	139 mg/dL	<200mg/dl
High-density Lipoprotein	39 mg/dL	>60mgl/dl
Low-density Lipoprotein	77 mg/dL	<100mg/dl
Triglyceride	113 mg/dL	<150mg/dl

Transthoracic echocardiography demonstrated concentric left ventricular hypertrophy (LVH). Systolic function was preserved with a calculated left ventricular ejection fraction (LVEF) of 59%, while diastolic assessment indicated grade 1 left ventricular diastolic dysfunction. There was no significant valvular disease. A diagnosis of non-ST-elevation myocardial infarction (NSTEMI) was made with a Thrombolysis in Myocardial Infarction (TIMI) risk score of 3. The patient was planned for early invasive strategy with a coronary angiogram.

Left heart catheterization was performed via a transfemoral approach using moderate sedation. Judkins left 4 (JL4) and Judkins right 4 (JR4) diagnostic coronary catheters were used for diagnostic coronary angiography. During the procedure, the patient experienced chest discomfort with each contrast injection into the left coronary system. Concurrently, continuous cardiac monitoring revealed a self-terminating episode of ST-segment elevation localised to lead V1. This response was not seen with the right coronary angiogram. Left ventriculogram performed with a 6 French pigtail catheter showed preserved LVEF estimated at 55%, consistent with the echocardiographic findings. 

**Figure 2:**
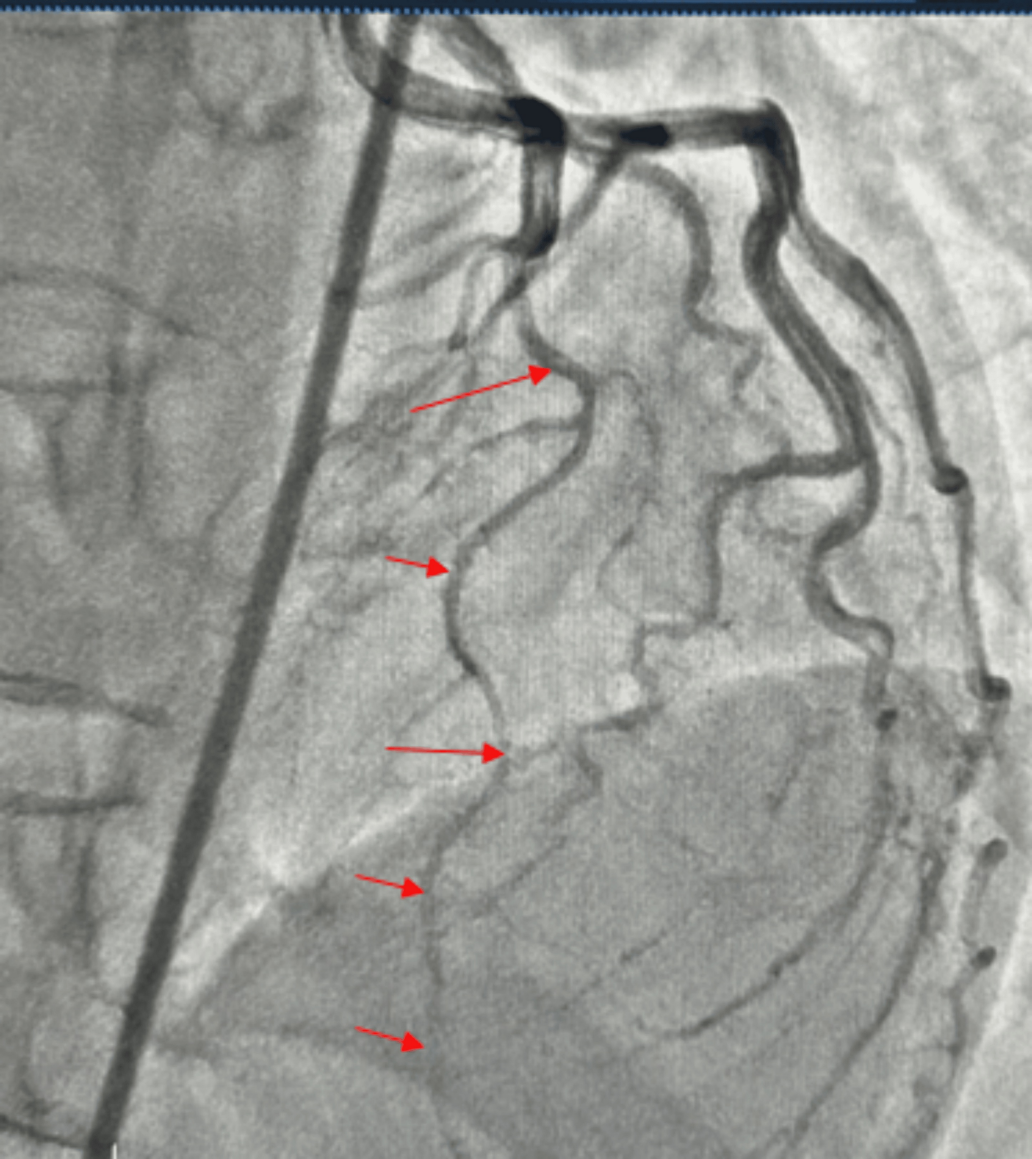
Angiogram showing vasoconstrictive segments of the left anterior descending artery (red arrows).

**Figure 3:**
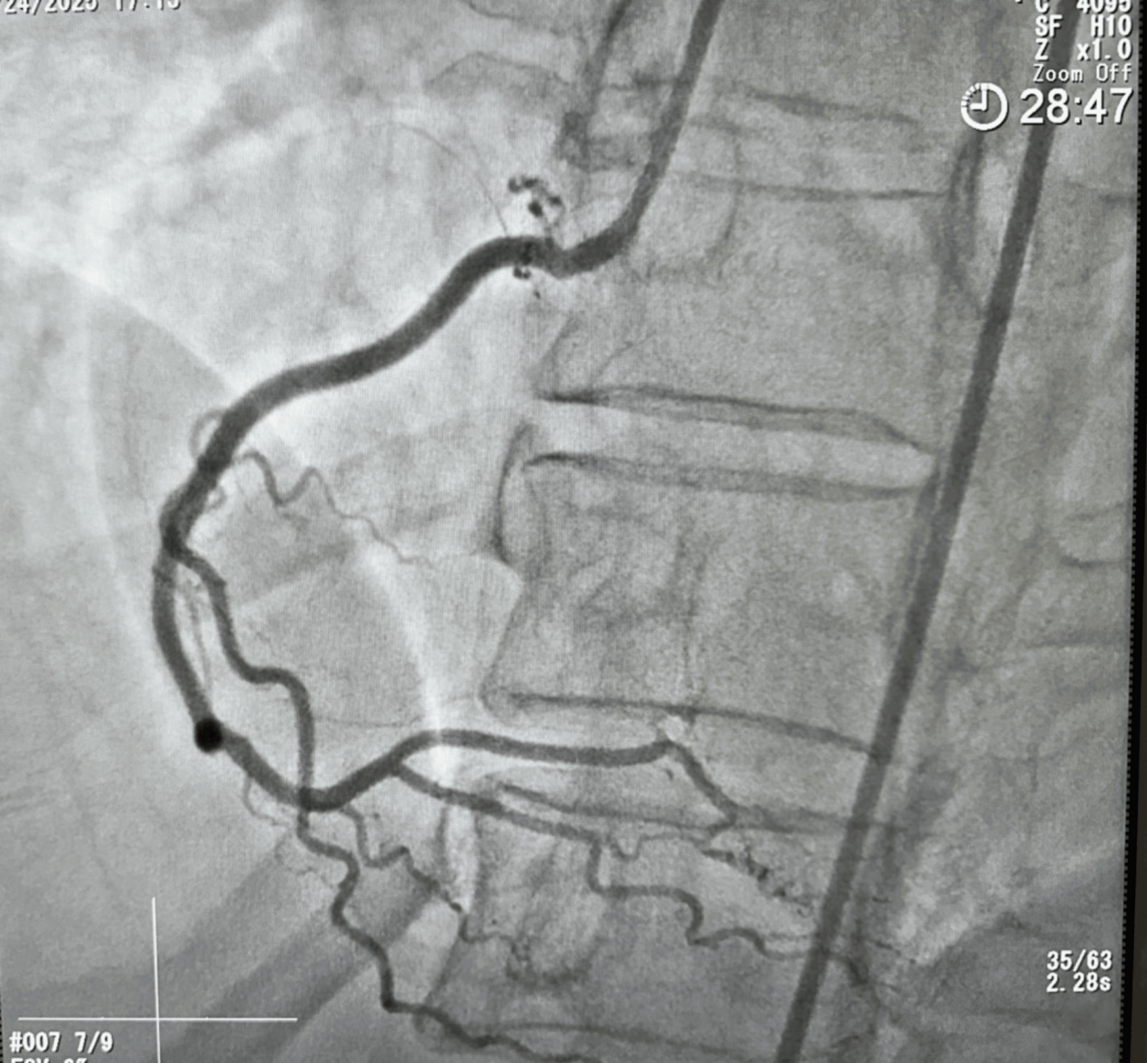
Angiogram showing the right coronary artery (RCA) with a mild proximal disease.

**Figure 4:**
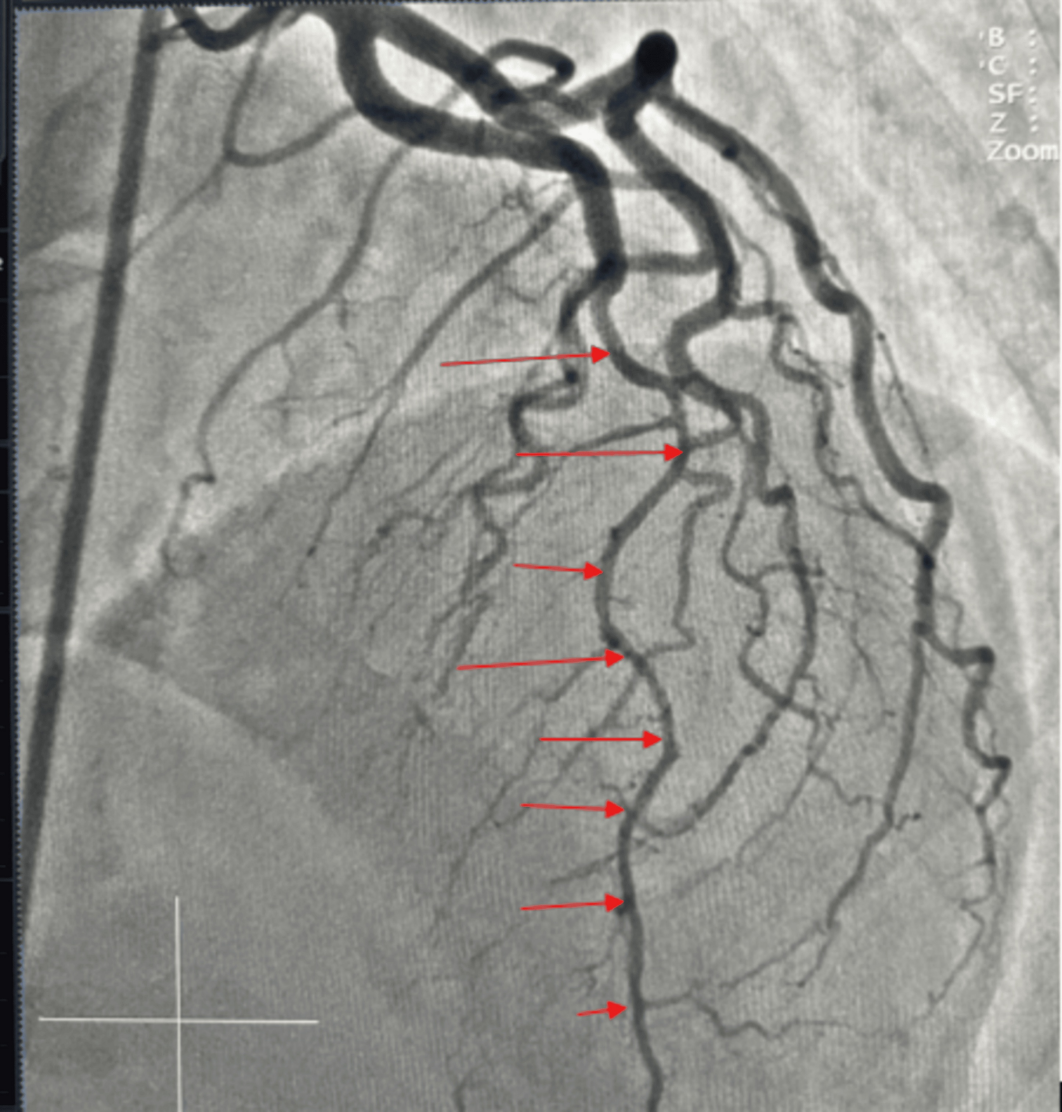
Angiogram showing resolution of spasm at the mid and distal segments of the left anterior descending artery (red arrows).

Coronary angiogram revealed a right dominant circulation. The left main coronary artery (LM), left circumflex artery (LCX) (Figure 2), and right coronary artery (RCA) (Figure 3) were angiographically normal (Figure 3) with no areas of spasm. The left anterior descending artery (LAD) demonstrated a normal proximal calibre, with vasospasm involving the mid and distal segments after take-off of a first diagonal branch (Figure 2). The LAD was free of any significant stenosis. She received 200 mcg of intracoronary nitroglycerin with improvement in spasm of the mid and distal segments (Figure 4). She was discharged on oral long-acting nitrate and calcium channel blocker with sustained symptom resolution on follow-up visits.

RCA was a dominant, normal-caliber vessel giving rise to the right posterior descending artery (RPDA) and right posterolateral (RPL) branches. The RCA had mild proximal disease (Figure 3).

## Discussion

The Coronary Vasomotion Disorder International Study Group (COVADIS) defines coronary artery vasospasm as a complete or near-complete (at least 90% occlusion), which manifests spontaneously or is induced by a stimulus, resulting in chest pain and concurrent ischemic changes observed on an electrocardiogram [14]. This definition describes succinctly what we observed in our patient.

In contemporary clinical practice, patients presenting with typical angina are found to have a spectrum of coronary anatomy, ranging from preserved coronary anatomy to various degrees of obstructive and non-obstructive atherosclerosis. There is a growing recognition of non-obstructive coronary artery disease, which is identified in approximately 50% of patients with stable angina and 57% of those presenting with acute coronary syndrome [15]. CAV remains the predominant etiology for ischemia in this context, underscoring the importance of understanding its pathophysiology [15].

The etiology of CAV is considered multifactorial, driven by a synergistic interaction among autonomic, endothelial, and inflammatory mechanisms. A primary hypothesis implicates autonomic nervous system dysregulation, where high vagal activity and acetylcholine release can induce spasm at rest, while subsequent catecholamine release from sympathetic activation may perpetuate ischemic episodes [16]. Endothelial dysfunction is a cornerstone of the disease process, often characterized by deficient nitric oxide (NO) bioavailability due to dysfunctional endothelial nitric oxide synthase (eNOS). This deficiency impairs endothelium-dependent vasodilation in response to agonists like serotonin and histamine, thereby promoting unopposed vasoconstriction [17]. Furthermore, oxidative stress plays a significant role; oxygen free radicals contribute to endothelial injury via NO degradation and direct vasoconstriction, particularly in the coronary microvasculature [18]. Genetic predispositions, such as aldehyde dehydrogenase (ALDH2) deficiency, exacerbate this oxidative stress by leading to toxic aldehyde accumulation, especially in individuals who consume alcohol [19]. Systemic inflammation, characterised by the elevation of pro-inflammatory biomarkers such as hs-CRP and interleukin-6, is also implicated in the pathogenesis of CAV [20]. Notably, diabetes mellitus persists as a particularly potent risk factor, often exerting a stronger influence on CAV risk than hs-CRP alone [20].

The diagnostic gold standard for CAV remains coronary angiography augmented with provocative testing (e.g., with acetylcholine or ergonovine). A positive test is defined as the reproduction of the patient's typical angina, accompanied by ischemic ECG changes and a significant reduction in coronary artery diameter [11]. Unfortunately, these agents are not available in some developing countries and were not available at our centre. Additional diagnostic metrics include the observation of elevated coronary arterial lactate levels concurrent with reduced coronary blood flow, which corroborates ischemia during spasm [21]. High-resolution intracoronary imaging, encompassing intravascular ultrasound (IVUS) and optical coherence tomography (OCT), can provide structural insights into the vessel wall during spasm, while cardiac magnetic resonance imaging (CMRI) is invaluable for differentiating CAV from other causes of MINOCA, such as myocarditis or takotsubo cardiomyopathy [22]. In our patient, however, normalization of the vasoconstrictive segments following administration of a potent vasodilatior, nitrolycerin, confirmed the diagnosis.

The management of CAV involves acute termination of episodes and long-term prevention. Sublingual or intravenous nitrates remain first line for acute symptomatic relief. Long-term therapy centers on high-dose calcium channel blockers (CCBs), with non-dihydropyridine agents (e.g., diltiazem, verapamil) often preferred for their combined vasodilatory and heart rate-lowering effects. Among dihydropyridines, benidipine has demonstrated superior efficacy in some studies [23]. Adjunctive therapies include statins to improve endothelial function, low-dose aspirin, magnesium supplementation, and antioxidants such as vitamins C and E to mitigate oxidative stress [24]. It is paramount to avoid non-selective beta-blockers, which can exacerbate vasospasm by allowing unopposed alpha-adrenergic activity; however, the highly selective beta-1 blocker nebivolol may be tolerated due to its NO-mediated vasodilatory properties [23].

## Conclusions

This case underscores the need for heightened clinical vigilance with a reminder that CAV can occur in patients without classic atherosclerotic risk profiles. In patients presenting with ischemic symptoms despite non-obstructive coronary arteries, a high clinical suspicion for coronary vasospasm is essential.

Therapeutic management centers on vasodilators, including nitrates and calcium channel blockers. Enhancing outcomes in CAV necessitates increased global awareness, uniform diagnostic standards, and therapies addressing the fundamental mechanisms of coronary hyperreactivity.
